# Brain death debates: from bioethics to philosophy of science

**DOI:** 10.12688/f1000research.109184.2

**Published:** 2022-06-20

**Authors:** Alberto Molina Pérez

**Affiliations:** 1 Institute for Advanced Social Studies, Spanish National Research Council (IESA–CSIC), Cordoba, 14004, Spain

**Keywords:** Death criteria, Brain death, Bioethics, Epistemology, Philosophy of Science, Functions, Irreversibility, Uniform Death Determination Act

## Abstract

50 years after its introduction, brain death remains controversial among scholars. The debates focus on one question: is brain death a good criterion for determining death? This question has been answered from various perspectives: medical, metaphysical, ethical, and legal or political. Most authors either defend the criterion as it is, propose some minor or major revisions, or advocate abandoning it and finding better solutions to the problems that brain death was intended to solve when it was introduced. Here I plead for a different approach that has been overlooked in the literature: the philosophy of science approach. Some scholars claim that human death is a matter of fact, a biological phenomenon whose occurrence can be determined empirically, based on science. We should take this claim seriously, whether we agree with it or not. The question is: how do we know that human death is a scientific matter of fact? Taking the philosophy of science approach means, among other things, examining how the determination of human death became an object of scientific inquiry, exploring the nature of the brain death criterion itself, and analysing the meaning of its core concepts such as “irreversibility” and “functions”.

## Introduction

More than 50 years after the neurological determination of death—also known as “brain death”—was admitted as a new criterion of death ( Beecher *et al.*, 1968), and despite its broad acceptance in medical practice ( Wahlster *et al.*, 2015; Lewis *et al.*, 2020b; Greer *et al.*, 2020), brain death continues to raise confusion ( Dalle Ave & Bernat, 2018; Rodríguez-Arias *et al.*, 2020), disagreement among scholars ( Joffe *et al.*, 2007, 2012; Youngner *et al.*, 1989), and opposition among the general public ( Skowronski *et al.*, 2021; Shah *et al.*, 2015). Brain death has been and still remains debated. In 2018, the Harvard School of Medicine organised a three-day conference on current controversies related to determining death ( Hastings Center Report, 2018). In 2020, the World Brain Death Project published consensus recommendations on determination of brain death ( Greer *et al.*, 2020). The same year, the US Uniform Law Commission (ULC) appointed a Study Committee on updating the 1981 Uniform Determination of Death Act (UDDA), which is the legal statute adopted in more than 40 states in the United States of America and which has had a significant influence in the laws throughout the world. In 2021, more than 100 scholars endorsed the Statement in Support of Revising the UDDA and in Opposition to a Proposed Revision ( Shewmon, 2021).

According to the current neurological criterion, as defined in the UDDA and other laws around the world, death is determined by the “irreversible cessation of all functions of the entire brain, including the brainstem”. Recent proposals to revise this criterion focus on the cessation of a limited number of functions, especially consciousness and some brainstem functions, including spontaneous respiration, and require the permanent ( *i.e. will not* reverse)—but not necessarily irreversible ( *i.e. cannot* reverse)—cessation of these functions ( Gardiner *et al.*, 2020; Lewis *et al.*, 2020a).

Foreseeable advances in medicine and neurosciences may completely challenge our current concept of human death by opening yet unknown possibilities to restore or reactivate consciousness, cognition, and other brain functions. Current and future technology, such as brain–computer interfaces ( Kübler, 2019; Abdalmalak *et al.*, 2020) linked to brain-stimulation technology ( Xia *et al.*, 2019; Fox *et al.*, 2020; Hakon *et al.*, 2020) and machine learning ( Iturrate *et al.*, 2020), may allow us to detect brain activity and function unnoticed today ( Owen, 2019), and to artificially restore some brain functions, for example through neural stem cell transplantation therapy ( Otsuki & Brand, 2018; Zhang *et al.*, 2019) or other therapeutic interventions ( Thibaut *et al.*, 2019), thus challenging the irreversibility of death ( Brummitt, 2018).

In 2019, researchers recirculated pigs’ brains, through a device called BrainEx, four hours after their decapitation ( Vrselja *et al.*, 2019). Glial cells were still able to maintain their inflammatory responses and neurons were responding to depolarizing current stimulation. Would such a device be able to restore human brain activities in similar conditions, stretching our knowledge on irreversibility of seemingly lost brain function? If so, would these patients be considered alive? It depends on whether those neural activities are mere activities or if they exert specific functions, like cognition or perception. Indeed, human death is usually determined by the irreversible cessation of either respiratory and circulatory functions, or all brain functions ( President’s Commission, 1981), while simple neuronal activities are ignored. However, the notions of activity and function are not clearly defined in the literature, and these notions are often used interchangeably, while their distinction may be key to define death ( Shemie *et al.*, 2014; Greer *et al.*, 2020).

In transplantation medicine, a clear and unambiguous determination of death is mandatory. Indeed, one of the main ethical norms in transplantation is the dead donor rule ( Dalle Ave *et al.*, 2020), which states that an individual should be declared dead before the procurement of any vital organs. This norm ensures that individuals will not be killed for transplantation purposes, and that they won’t suffer during organ recovery. What would happen if there were no clear criteria for determining death? Are certainty and universality required for the determination of death or could some degree of uncertainty and diversity be acceptable?

Advances in medical technology may enable perception, motricity, cognition, or communication, to be artificially prolonged beyond a state in which they would normally be irreversibly destroyed. What would that mean for our understanding of what it is to be a human person? What kind of quality of life would such technology offer? Would that life be worth living? How would it impact social justice and equality? How would it affect personal identity and human rights? What would be the future of humankind if, in our quest to surmount mortality, we could eliminate the very concept of death through technology and neurosciences ( Sandberg *et al.*, 2008; Bamford & Danaher, 2017; Harari, 2018)?

These questions are of a philosophical nature and belong, in particular, to the domain of moral philosophy. To this day, most academic debates around brain death have been and are bioethical. However, I believe that a different philosophical approach can shed light on old controversies and help either settle open disputes or, on the contrary, raise new questions. I am talking about the philosophy of science. Death criteria are supposed to have an epistemic or scientific value. Some scholars claim, and most physicians certainly agree, that human death is a matter of fact, a biological phenomenon whose occurrence can be determined empirically, based on scientific knowledge and methods, and that medicine has epistemic authority over it. We should take this claim seriously, whether we agree with it or not, and analyse it from the perspective of the philosophy of science.

## 50 years of bioethical controversies around death determination

Since it was introduced in 1968, the brain criterion of death is at the centre of a heated debate that has produced a vast and complex literature. The debate among bioethicists has had some key recurring features ( Belkin, 2018): first and foremost, argument over alleged flaws in the conceptual logic and consistency of the rationale for equating brain death with human death; second, efforts to fix perceived limitations of brain death-based practices to optimize transplantation; and third, a basic unease provoked by the experience of using the criteria and managing a warm and heart-beating body in a state previously known as “irreversible coma.”

With regard to the second feature, the 2018 special Hastings Center Report illustrates the central role played by the dead donor rule in bioethical debates (see also: Arnold & Youngner, 1993; Veatch, 2004; Nair-Collins *et al.*, 2015; Rodríguez-Arias, 2018; Dalle Ave *et al.*, 2020).

With regard to the first feature, one of the first and still open controversies concerns the nature of the justification of introducing brain death as a criterion of death, some claiming it had been initially proposed to solve practical and moral problems, including the opportunity of recovering organs without violating the “dead donor rule” ( Pernick, 1999; Rodríguez-Arias, 2017).

In 1981, Bernat, Culver and Gert proposed the “whole-brain” concept of death, a scientific and philosophical justification based on the idea that life requires the integration of the organism and that the brain is the organ responsible for its integration ( Bernat *et al.*, 1981). Apart from the UK and a handful of nations, most developed countries soon adopted the whole-brain rationale ( Wijdicks, 2002). However, Bernat and colleagues’ claim that individuals with a cessation of all brain functions are not integrated organisms but merely a group of artificially maintained subsystems has been challenged repeatedly and decisively ( Youngner & Bartlett, 1983; Gervais, 1986; Halevy & Brody, 1993; Lizza, 1993; Seifert, 1993; Veatch, 1993; Taylor, 1997; Truog, 1997; Brody, 1999; Halevy, 2001; Potts *et al.*, 2001; Shewmon, 1998, 2001; Byrne & Weaver, 2004; Zamperetti *et al.*, 2004; Joffe, 2007; Shemie *et al.*, 2014; Brugger, 2016; Verheijde *et al.*, 2018). As a consequence, some advocates of the brain criterion have proposed refined versions of this rationale in terms of “organism-as-a-whole” ( President’s Council, 2008; Moschella, 2019; Bernat, 2019; Huang & Bernat, 2019; Omelianchuk, 2021), whereas critics have proposed alternatives in terms of “embodied consciousness” ( Veatch, 2005; Veatch & Ross, 2016), personhood ( Green & Wikler, 1980; Bartlett & Youngner, 1988; Lizza, 2006), or homeostasis ( Nair-Collins, 2018). Most of these propositions are compatible with a single brain-based criterion of death, although for different reasons and with varying implications for the boundary between life and death.

A different way of addressing the issue of determining death is to focus on its intrinsically legal, ethical, and political nature, by arguing that brain death is a legal fiction ( Shah *et al.*, 2011), advocating for a pluralistic policy that would allow stakeholders to choose among several definitions of death ( Veatch, 1976; Bagheri, 2007; Molina-Pérez *et al.*, 2008; Ross, 2018), requiring consent for brain death testing ( Berkowitz & Garrett, 2020), and calling for an open public conversation on end-of-life practices ( Youngner & Arnold, 2001; Rodríguez-Arias & Veliz, 2013).

## Taking the philosophy of science approach to human death

Death debates focus on a single question: Are death criteria, and especially the brain criterion, *good* criteria? They may be good in a scientific or medical sense because patients declared dead according to these criteria are actually dead, although there may be false positives and false negatives ( Bernat & Dalle Ave, 2019). They may be good in a moral or policy sense because, for instance, they allow practices that are ethically and socially valuable, such as organ procurement. They may be good for a combination of reasons. These reasons have their advocates and opponents, both committed to truth and quality scholarship and based on good evidence and well-considered scientific and/or philosophical arguments. Almost all authors have either defended the death criteria, proposed some minor or major revisions and improvements of the criteria, or plead to abandon them—especially the brain criterion—and promoted better solutions to the problems these criteria were meant to solve.

Regardless of whether death criteria are true or accurate, or whether they are good policy, we should take a step back to examine the issue of “determining death” as an object of scientific inquiry by itself. This is the philosophy of science approach that I am advocating. Taking this approach means examining how the determination of human death became a scientific issue and why medicine claims epistemic authority over it. It means asking, for example, whether death criteria are heuristics or definitions, and whether the determination of the patient’s death is the conclusion of a syllogism. To adopt this approach is also to analyse the meaning of the core concepts of death criteria, such as “irreversibility” and “functions”. Ideally, the answers to these questions could be shared by most scholars, regardless of their position on the issue of brain death.

Analyses of death definitions and criteria that take science seriously are scarce ( Nair-Collins, 2015). In addition to the question of whether the main justification for brain death is rather scientific/epistemic or moral/practical, debates with a more philosophy of science orientation have revolved around two central questions: the nature of death as an event or a process ( Morison, 1971; Pallis, 1983), and the requirement of irreversibility (as opposed to permanence) for the loss of functions ( Bernat, 2010; Dalle Ave & Bernat, 2016; Gardiner & McGee, 2017). To my knowledge, few other publications have adopted what I consider a philosophy of science approach, with some notable exceptions ( *e.g.*: Walton, 1981; Chiong, 2005; Nair-Collins, 2015; Meier, 2020).

Taking this approach may help settle old debates and also bring novel insights. For example, death is characterised in medicine and described or defined in most legislations as the irreversible cessation of specific functions: either circulatory and respiratory functions or all brain functions. However, the meaning of the concepts used, especially the concept of function, requires clarification. These concepts are rarely defined in the medical literature and their interpretation varies between professionals. Hence, the consistent interpretation of the death criteria that rely on these concepts is not warranted, which may cause a lack of uniformity in death determination.

By applying to the criteria of death an analysis similar to the one used in the philosophy of biology to define biological functions ( Molina-Pérez, 2017), Anne Dalle Ave, James Bernat and I revealed that the current US law is conceptually inconsistent ( Molina-Pérez *et al.*, forthcoming). Indeed, the UDDA uses the phrase “cessation of functions” with two different and conflicting meanings for its two criteria. On the one hand, it means the cessation of spontaneous functions, i.e. the cessation of the organ’s spontaneous functioning. On the other hand, it means the cessation of either spontaneous or artificially supported functions. We also showed that this inconsistency in the UDDA—and other laws throughout the world that acknowledge both criteria—derives from the conceptual assumptions underlying James Bernat’s 1981 “Whole-Brain” rationale for equating brain death with death. By conducting a logical deconstruction of the rationale, we showed that its premises are false and that, therefore, its conclusions cannot be drawn.

This type of analysis leads to policy recommendations and ethical considerations. After exploring possible ways to address the inconsistency, we found only two viable options: one is to keep the law as it is while admitting that death is a legal fiction, and the other is to pick a single criterion of death, either a circulatory-respiratory criterion, which would imply that “brain dead” patients maintained in the ICU are not dead, thus disrupting organ procurement (DBD) programmes, or a single brain criterion, which may affect medical practice and hinder programmes of organ recovery after circulatory death (DCDD) (see Rodríguez-Arias *et al.*, 2013; Dalle Ave *et al.*, 2016; Ortega-Deballon & Rodríguez-Arias, 2018).

In order to better assess death determination criteria, further analyses of the concept of function are needed. On the one hand, brain functions are at the intersection of three different but related scientific fields: biology, psychology, and neurology. While functions in general have been very much discussed in philosophy of biology ( Garson, 2016; Molina-Pérez, 2017) and to some extent in philosophy of psychology ( Ariew *et al.*, 2002; Parot, 2008), they have received much less attention from either physicians or philosophers of medicine ( Roux, 2014; Shewmon & Verheijde, 2020). A distinction needs to be made between the brain’s functions, as considered by physiology, and the brain’s functionality, as considered by neuroscience ( Northoff & Tumati, 2019). In the context of neurophilosophy and brain-computer interfaces, brain’s functionality requires visible and coordinated behavioural responses in reference to specific environmental contexts. In other words, brain functions are not merely considered as physiological mechanisms, but also viewed in terms of whether they serve their “intended behavioural purpose”.

On the other hand, as critical care medicine keeps pushing forward its resuscitation capabilities, we still do not know where to find—and whether there are—absolute limits to the reversibility of functional losses. The cessation of brainstem functions, such as the initiation of respiration, can now be reversed with life-support technologies such as mechanical ventilation. This means that, although the spontaneous control of these functions by the brain is lost, the functions themselves can still be executed and controlled by artificial means ( Meier, 2020; Molina-Pérez *et al.*, forthcoming). Future advances, including BCIs, might help support, restore or replace not only brainstem functions, but also those necessary for cognition, and may, consequently, alter the threshold of irreversibility. This raises the question of the limits and meaning of irreversibility in the determination of death.

The cessation of functions can be interpreted in at least three different ways: as absolutely irreversible, relatively irreversible, or permanent ( Tomlinson, 1993; Bernat, 2010). Absolute irreversibility means that the cessation of functions cannot be reversed under any circumstances, regardless of any present or future medical and technological interventions. It could be understood in the sense of a physical or biological impossibility to, past some point, reverse the dying process. Relative irreversibility means that the cessation of functions cannot be reversed in some context, but might be reversed in a different one. It could be understood as an incapacity to reverse the loss of functions because of a lack of human competence, technical means, resources, etc. Most circulatory and respiratory arrests were irreversible in the 19th century, before the development of resuscitation techniques, but can now be reversed because these means now exist. Relative irreversibility thus depends on when and where the capacity to reverse the loss of functions is available and feasible, which in turn depends on other considerations, including the ethical, social, economic, and political framework. However, it is not always clear which of these two interpretations of the notion of irreversibility (absolute or relative) is used in current definitions and criteria of death. Finally, permanence means that the loss of functions will not reverse spontaneously and that, although we would be capable of reversing it, we will not do so. This may occur for instance to respect the patient’s living will.

## Data availability

No data are associated with this article.
