# Supplementary material for: Brain death debates: from bioethics to philosophy of science
Source: F1000Res. 2022 Jun 20;11:195. Originally published 2022 Feb 16. [Version 2] doi: 10.12688/f1000research.109184.2 (PMC9253658; doi:10.12688/f1000research.109184.2)
Supplement: Peer review history

# Peer review: Reviewer response for version 2

**DOI:** 10.5256/f1000research.126017.r141312

**Published:** 2022-07-04

**Authors:** Michael Nair-Collins

**Affiliations:** 1 Department of Behavioral Sciences and Social Medicine, Florida State University College of Medicine, Tallahassee, FL, USA

No further comments. I congratulate the author on a thoughtful and well-informed manuscript.

Is the topic of the opinion article discussed accurately in the context of the current literature?

Yes

Are arguments sufficiently supported by evidence from the published literature?

Yes

Are all factual statements correct and adequately supported by citations?

Yes

Are the conclusions drawn balanced and justified on the basis of the presented arguments?

Yes

Reviewer Expertise:

Bioethics, philosophy, brain death

I confirm that I have read this submission and believe that I have an appropriate level of expertise to confirm that it is of an acceptable scientific standard.

# Peer review: Reviewer response for version 2

**DOI:** 10.5256/f1000research.126017.r141313

**Published:** 2022-06-27

**Authors:** Milena Maglio, Vivien García

**Affiliations:** 1 Clinical Ethics Center, Greater Paris University Hospitals (Assistance Publique-Hôpitaux de Paris), Paris, France; 2 SPHERE, Université Paris Cite, Paris, France; 3 École de Santé Publique de Nancy, Université de Lorraine , Nancy, France

No further comments

Is the topic of the opinion article discussed accurately in the context of the current literature?

Yes

Are arguments sufficiently supported by evidence from the published literature?

Yes

Are all factual statements correct and adequately supported by citations?

Yes

Are the conclusions drawn balanced and justified on the basis of the presented arguments?

Yes

Reviewer Expertise:

Milena Maglio: moral philosophy, bioethics and clinical ethics. Vivien García: moral and political philosophy, ethics and philosophy of technology.

We confirm that we have read this submission and believe that we have an appropriate level of expertise to confirm that it is of an acceptable scientific standard.

# Peer review: Reviewer response for version 1

**DOI:** 10.5256/f1000research.120659.r123955

**Published:** 2022-03-31

**Authors:** Milena Maglio, Vivien García

**Affiliations:** 1 Clinical Ethics Center, Greater Paris University Hospitals (Assistance Publique-Hôpitaux de Paris), Paris, France; 2 SPHERE, Université Paris Cite, Paris, France; 3 École de Santé Publique de Nancy, Université de Lorraine , Nancy, France

In “Brain death debates: from bioethics to epistemology”, Alberto Molina Pérez recalls the still controversial character of the neurological determination of death. He also maintains that “to this day, most academic debates around brain death have been and are bioethical”. To support this claim, he draws on extensive literature on the topic; this is undoubtedly one of the great qualities of his contribution. But the main thesis of this opinion article goes further, Molina Pérez argues for a shift of the debate toward “epistemology or, to be more accurate, … philosophy of science”. More exactly, he suggests examining “the determination of death as an object of scientific inquiry”. Aiming at illustrating the relevance of the change of approach he pleads for, Molina Pérez briefly analyses two core concepts of death criteria — “irreversibility” and “functions” — and shows that each of them bear meaning which are different and possibly conflicting.

 The main strength of the paper lies in this demonstration, in particular, in the analysis of the concept of “functions”. The distinction between “brain functions” and “brain functionality” is quite convincing and original. Although promising, the analysis of the concept of “irreversibility” is not so clear. Here come the first suggested revision. On page 3 (§2), the author refers to a distinction between “permanent” and “irreversible” cessation of “functions”. In the first case, he says, the cessation “will not reverse” but it is “not necessarily irreversible”. In the second case, the cessation “cannot reverse”. However, on page 5 (last §). Molina Pérez presents 3 possible interpretations of “irreversibility”: “as either absolute, relative, or permanent”. Presented this way, the permanent cessation of a functions seems to be one type of irreversibility. Moreover, only 2 of the 3 terms are explained, so it is not clear if relative and permanent “irreversibility” can be taken as synonyms. We suggest that the author clarify the formulations of these two paragraphs.

 Another possible criticism (which leads to the suggestion of a second minor revision) could be that the author raises many interesting questions without addressing them, i.e: “What a death criterion is: is it a heuristic or a definition? Is it descriptive, stipulative or performative? Is it a rule of inference, and if so, what is its logical structure? For example, is the determination of the death of an individual the conclusion of a syllogism?”

 This is problematic because:

1. these questions are clearly related to the main goal of this paper: “examining how the determination of human death became a scientific issue and why medicine claims epistemic authority over it”;
2. according to the author, here lies all the interest of his “epistemological or philosophy of science approach”.

If the author has already addressed these questions elsewhere, or plans to do so, he should at least provide references. If not, he could reduce the number of questions and focus on the relevant ones for the present article.

 As pointed out in Michael Nair-Collins' review, Molina Pérez should make clear what he means by “epistemology” and “philosophy of science”. As non-native English speakers, and academics trained in continental philosophy, our understanding of how Molina Pérez uses these words is probably different from Nair-Collins' one. To prevent misunderstandings (and this is the third revision suggested), the author should definitely clarify his take on the matter.

 Beyond these terminological concerns, and without suggesting any revision this time, we would like to stress that the core thesis of the paper — the need for a shift “from bioethics to epistemology” — is not self-evident. As a matter of fact, after reporting the outcomes of a forthcoming study, Molina Pérez argues that the “type of analysis [he supports] leads to policy recommendations and ethical considerations”. In a provocative way, one could say that the author finally moves from “epistemology” to bioethics, or assert that this epistemological “digression” fundamentally serves (normative) ethical purposes.

 The brain death debates could certainly benefit from taking more seriously the “epistemological” approach. But the latter will hardly eliminate, in particular in the medical field, a haunting ethical question: why do we value human life? This is not a mere theoretical problem. In some medical practices, in end-of-life decisions, before the determination of death comes into play (and whatever criteria we choose for this purpose) this question frequently reappears, either implicitly or explicitly. For example, in Controlled Donation After Cardiac Arrest — even if the Dead Donor Rule remains a fundamental moral norm for organ retrieval — the issues related to futile treatments and the decision to withdraw life-sustaining treatments can be far more relevant than the definition of death.

Is the topic of the opinion article discussed accurately in the context of the current literature?

Yes

Are arguments sufficiently supported by evidence from the published literature?

Yes

Are all factual statements correct and adequately supported by citations?

Yes

Are the conclusions drawn balanced and justified on the basis of the presented arguments?

Yes

Reviewer Expertise:

Milena Maglio: moral philosophy, bioethics and clinical ethics. Vivien García: moral and political philosophy, ethics and philosophy of technology.

We confirm that we have read this submission and believe that we have an appropriate level of expertise to confirm that it is of an acceptable scientific standard, however we have significant reservations, as outlined above.

# Response

**Published:** 2022-04-07

**Authors:** Alberto Molina Pérez

**Affiliations:** Spanish National Research Council, Spain

**Comment:** *“the analysis of the concept of ‘irreversibility’ is not so clear”*

**Response:** I appreciate your comment on the lack of clarity of the term “irreversibility”. In the first paragraph you mention, the focus is on the requisite of permanence (i.e. will not reverse) as opposed to irreversibility (i.e. cannot reverse). Although briefly explicated, both terms remain quite vague but I would prefer not to enter into further details there. In the second paragraph you mention, further clarification is indeed necessary. I have made a series of changes to better explain absolute irreversibility, relative irreversibility, and permanence.

**C:** *“Another possible criticism…”*

**R:** With regard to your comment on unaddressed questions, my objective was to show a sample of the questions that can be asked from a philosophy of science perspective and for which, as far as I know, no one has yet proposed an answer, neither have I. This article is a way to draw the attention of researchers like you to a field of research that is still underexplored and to lay the foundations for future work; a work that will require the collaboration of many.

 To avoid the accumulation, I have rephrased the passage as follows: “It means asking, for example, whether death criteria are heuristics or definitions, and whether the determination of the patient’s death is the conclusion of a syllogism.”

**C:** *“Molina Pérez should make clear what he means by ‘epistemology’ and ‘philosophy of science’…”*

**R:** To avoid any confusion, I have removed the term “epistemology”.

**C:** *“Beyond these terminological concerns…”*

**R:** It is true that after conducting a conceptual and logical analysis of death criteria from a philosophy of science perspective, my colleagues and I moved back to bioethics. I sincerely hope that this back and forth may prove useful to address normative questions. However, I would not say that the epistemological approach is a digression at the service of ethical purposes. In my opinion, these are two autonomous and complementary ways to approach and address some of the issues raised by the determination of human death.

**C:** *“The brain death debates could certainly benefit from taking more seriously the “epistemological” approach. But…”*

**R:** I agree that some end-of-life ethical issues, such as the dead donor rule and the decision to withdraw life-sustaining treatments, are independent from the epistemic issues raised by the definition and determination of death. I believe it is important to distinguish, as much as possible, facts from values, ‘is’ from ‘ought’. One of the criticisms formulated against brain death is that it is a way of disguising normative questions under the appearance of a factual question. If we are not careful, the same strategy could be used for other end-of-life issues.

# Peer review: Reviewer response for version 1

**DOI:** 10.5256/f1000research.120659.r123954

**Published:** 2022-03-21

**Authors:** Michael Nair-Collins

**Affiliations:** 1 Department of Behavioral Sciences and Social Medicine, Florida State University College of Medicine, Tallahassee, FL, USA

In “Brain death debates: from bioethics to epistemology,” Alberto Molina Pérez argues that the debates on brain death have been characterized by a partisan nature: authors are, roughly, either for or against brain death as an appropriate criterion for death. In place of partisanship, Molina Pérez suggests a non-partisan, epistemological or philosophy of science-based approach to the topic, which would involve examining how the nature of death became an object of scientific inquiry, exploring the brain death criterion, and analyzing the core concepts of “irreversibility” and “function”.

 Overall, I think the article has the potential to contribute something of value to the brain death literature. Its key point – that the brain death literature can benefit from an examination rooted in philosophy of science – is well-taken. Is death something that can be studied empirically, is it a matter of physiology? Can there be an objective, rationally defensible answer to the question of what is human death, as a matter of physiology? Alternatively, is the nature of death necessarily a “social construction” or fundamentally a matter of agreement rather than discovery? Are all biological categories necessarily vague? Are pluralistic concepts always appropriate, such as how, some argue, the species concept is appropriately pluralistic and different species concepts can be marshalled for different scientific purposes – and so too for the concept of function, irreversibility, and death? And so on… These are all fundamentally situated in theories from the philosophy of science and philosophy of biology, and as such, these areas of research have much to offer the bioethical debates about brain death.

 My main suggestions for revision involve the concept of partisanship and the terminology of “epistemology” vs. “philosophy of science”.

 To suggest partisanship is to suggest a strong commitment to a cause (or political party), which is grounded in noncognitive reasons of affiliation with one’s “team”, rather than a commitment to truth and quality scholarship. This rendering of the debate seems to suggest that there aren’t any standards by which we might say that one view has better evidence and reasons in support of it; it also suggests that it is not the case that at least one side is, in fact, correct.

 Of course, brain death is a very longstanding debate, and it is very complex, drawing on metaphysics, philosophy of science, physiology, ethics, and much else besides, and it may well be true that we are unable to determine – as an epistemic matter – which side, if any, is actually right. But those considerations demand epistemic and moral humility. They do not lead quite so far as to suggest that this is a partisan issue of affiliation and persuasion, and that there aren’t any standards we could appeal to in evaluating distinct views. One could attempt to defend the view that all debates in scientific domains are characterized by something like incommensurability and as such, adherents are, in fact, partisan, and reason and evidence would not arbitrate such disputes. However I don’t think that broader thesis is being made here.

 Second, the notion that taking a bioethical approach leads to partisanship, while taking a philosophy of science approach is non-partisan, doesn’t make sense to me. Why would simply approaching the issues from one perspective render one partisan, but approaching them from a different perspective render one non-partisan? Furthermore, any conclusions drawn from a philosophy of science approach are going to have implications for questions regarding brain death anyway, which would seem to lead back to partisanship on this view anyway.

 Overall I don’t get the sense that the concept of partisanship does a lot of work in the paper. My recommendation is to remove it entirely, or if not, clarify its use in light of the above comments.

 Regarding the terms “epistemology” and “philosophy of science”, I am a little uncertain about how they are used here. My understanding is that epistemology is the study of knowledge, while philosophy of science is a field that studies many aspects of science and its practice, drawing on epistemology, metaphysics, formal logic, philosophy of language, and so on. I am certain that I would not be alone in this interpretation of these terms, so I would suggest just rephrasing in terms of “philosophy of science” throughout and removing “epistemology” to avoid creating unnecessary confusion for readers.

 What follows are a number of comments; this should be read simply as having a conversation, rather than any particular requests for revisions based on the text below. The author should make any changes they think important or interesting enough, but need not reply to every point.

 Some comments on the term “function”: It is stated in the paper that “the notions of activity and function are not clearly defined in the literature” (p. 3).  Perhaps their definitions are motivated, and bad definitions, but they are defined in the literature. The World Brain Death Project (Greer et al. 2020) and the International Guidelines for Determination of Death group (Shemie et al 2014) both explicitly define these terms, where “function” means something like “measurable on bedside neurological examination” and “activity” means “cellular phenomena recordable by technology”.

 Granted, I think that their definitions are bizarre, and clearly motivated by the goal of pretending that clinical practices in the diagnosis of brain death are consistent with the UDDA and the whole-brain criterion of death, when they are certainly not, since some brain function in the form of osmoregulation can continue in patients declared brain dead, and hence it is not true that “all functions of the entire brain” have ceased.

 It is interesting to ask about the field within which we should address the question of function (cf. p. 5 discussion of biology, psychology, neurology definitions of function). Quite obviously, in this context the field is clinical medicine. And in clinical medicine, physicians assess cardiac, renal, hepatic (etc.) function using laboratory tests and imaging technology. It would be absolutely bizarre to suggest that a physician cannot assess their patient’s kidney function by evaluating lab measurements of urine and blood, because this only shows “activity and not function”. It would be similarly bizarre to suggest that a physician cannot assess their patient’s heart function with an EKG because this reflects the electrical activity of cells “recordable by technology”. This is what Greer et al 2020 and Shemie et al 2014 are saying with their definitions. The purpose is to be able to claim is that osmoregulation is not a function because it cannot be assessed at the bedside (which isn’t true anyway – normal urine output signals osmoregulation and hypothalamic function).

 In any case, on any reasonable understanding of how physicians can and do assess the function of organs, there must be a variety of ways to do that, including bedside assessment, questionnaires, laboratory tests, imaging, and so on.

 Finally, the notion that “brain functions are not merely considered as physiological mechanisms, but also viewed in terms of whether they serve their intended behavioral purpose” (p. 5) seems incorrect, or to build in illicit assumptions, even within the context of neurophilosophy and brain-computer interface. First, any theory of function that makes it come out that all brain functions must have an overt, 3 ^rd^-person observable, behavioral manifestation does not seem right. There are many functions the brain is involved in that involve managing the homeostatic environment of the physiochemical milieu, even as simple as shunting blood from the periphery to the core, or causing blood vessels to constrict or dilate, and so on. In locked-in syndrome, the entire suite of normal cognitive functions are maintained, and occur without behavioral manifestation. Some of these cognitive functions become apparent through blinking, for sure, but not all. To assume that brain functions must have an overt behavioral manifestation seems to take an unjustified behaviorist approach to mentation.

 And then second, a function as understood within the context of natural selection would not have any “intended” purpose, behaviorally manifested or not.  A function is something that had some advantage in an environment of evolutionary origin, and was reproduced, and that is it, at least on this view. For example, the intrinsic automaticity of the sinoatrial node manifests a clear function in preserving the heartbeat, but it does not “intend” anything, nor does it have an “intended purpose”. Who could have intended such a purpose?

 On the dead donor rule: The manuscript states [death criteria] “may be good in a moral or policy sense because … they allow … organ procurement” (p. 4). This is a common thing to say, but I think it is confused. The criteria for death neither allow nor prohibit any policy or practice. In the case of organ procurement, it is the dead donor rule that prohibits organ procurement, not death criteria. If we as a society agree that donors should be dead first – we support the dead donor rule – then they should be dead. The criteria for what constitutes being dead cannot then be derived in such a way that it bypasses the entire point of the dead donor rule, which is to restrict organ procurement. If the dead donor rule matters, then the criteria for death must be independent of the dead donor rule itself, otherwise it becomes trivial, and is not a genuine constraint on behavior at all. On the other hand, if it’s ok to take organs from patients in state X, AND it turns out that patients in state X are still living, then it follows that the dead donor rule need not be adhered to. Either way, death criteria do not prohibit or allow anything.

 Nair-Collins does not defend the brain criterion (p. 4): “As a consequence, some advocates of the brain criterion …, or homeostasis (Nair-Collins 2018)”.

Is the topic of the opinion article discussed accurately in the context of the current literature?

Yes

Are arguments sufficiently supported by evidence from the published literature?

Yes

Are all factual statements correct and adequately supported by citations?

Yes

Are the conclusions drawn balanced and justified on the basis of the presented arguments?

Yes

Reviewer Expertise:

Bioethics, philosophy, brain death

I confirm that I have read this submission and believe that I have an appropriate level of expertise to confirm that it is of an acceptable scientific standard, however I have significant reservations, as outlined above.

# Response

**Published:** 2022-04-07

**Authors:** Alberto Molina Pérez

**Affiliations:** Spanish National Research Council, Spain

**Comment:** *“To suggest partisanship is to suggest a strong commitment to a cause…”*

**Response:** With regard to the use of the term “partisanship”, I really appreciate your comment because I was not aware that this term could be interpreted in this context as it is in the political sphere. This was not the intention. As you say, this rendering of the debate would suggest “that there aren’t any standards by which we might say that one view has better evidence and reasons in support of it” and “that it is not the case that at least one side is, in fact, correct”. However, this is not the way I see the debate; quite the contrary. On the one hand, I think that all parties involved are strongly committed to truth and quality scholarship, and that their views are based on good evidence and well-considered reasons. On the other hand, I also believe that it is possible to critically assess the distinct views and show that some of them are based on flawed, inconsistent or unsatisfying arguments from a scientific and/or philosophical perspective. To do so in a useful way, we need to agree on the standards of evaluation and on the meaning of the concepts used. Some disagreements may be based on the fact that the different parties do not speak the same language, although they think they do. Other disagreements may stem from the fact that the parties have different objectives or different interpretations of what a “good” criterion means to them, although they are not necessarily aware that this is the root of their disagreement. This is why I think a “neutral” approach is needed to be on the same page. This would not solve all disagreements, but it would enable more fruitful debates based on more solid ground.

**C:** *“Second, the notion that taking a bioethical approach…”*

**R:** I agree with you. However, (bio)ethics refers to well-founded standards of right and wrong that prescribe what we ought to do. In this case, the question is whether or not we should keep using brain death (as it is or some modified version of it) to determine human death. Some support using brain death because they consider it is a good/right criterion for varying reasons (scientifically, ethically, as a policy, etc.) while others oppose it because they consider it is a bad/wrong criterion (scientifically, ethically, as a policy, etc.).

 The approach I am advocating for in this article means taking a step back, setting aside the question of whether brain death is good/right or bad/wrong, and asking more general questions to understand the very nature of a (any) death criterion. 

 This is similar to your own proposition: “ *An objective and unbiased investigation of the biology of death is independent of, and should be undertaken prior to, an analysis of the normative questions engendered by debate about determination of death.*” (Nair-Collins 2015)

 Perhaps the main difference is that you emphasize the scientific questions about the biology of death and dying, which I totally agree with, whereas I emphasize the (more general?) philosophical questions about the science of death determination. In some cases, both approaches coincide. For example, with regard to the distinction between activities and functions, you show how the definition of “function” in terms of clinical observability by the IGDD group is an ad hoc strategy to rule out neuroendocrine functions (op. cit.). This is real philosophy of science and it's beautifully done. However, a more general question remains unanswered: how does/should medicine define functions and distinguish them from mere activities? I think it is (ideally) possible to come to an agreement on this issue regardless of where everyone stands on brain death. But it is true (hopefully) that the conclusions drawn from this—objective and unbiased, to use your words—approach of functions in medicine can have implications for the debates on brain death and the question of whether we should keep using it as is, revise it, or abandon it.

**C:** *“Overall I don’t get the sense that the concept of partisanship does a lot of work in the paper.”*

**R:** I have removed any reference to partisanship from the abstract and the text. I have also tried to emphasized that discrepancies between scholars are not based on mere opinions or ideological affiliations:

 “They [death criteria] may be good for a combination of reasons. These reasons have their advocates and opponents, both committed to truth and quality scholarship and based on good evidence and well-considered scientific and/or philosophical arguments.” (p.3)

 “ Ideally, the answers to these questions could be shared by most scholars, regardless of their position on the issue of brain death.” (p.3)

**C:** *“Regarding the terms ‘epistemology’ and ‘philosophy of science’…”*

**R:** I understand your confusion with regard to the use of the term “epistemology”. In France and in Spain, where I trained as a philosopher, “epistemology” and “history and philosophy of science” are used as synonyms. This is not the case elsewhere. In the first draft of the manuscript, I used “epistemology” alone because it felt more natural to me, then I added “or philosophy of science” to try to convey this equivalent meaning. Following your advice, to avoid any confusion for readers, I removed the term “epistemology”.

**C:** *“Some comments on the term ‘function’…”*

**R:** Definitions of activity and function are scarce and, when available, suboptimal (to say the least). I have included in the manuscript the two references you mention so that the reader can make their own opinion.

**C:** *“Granted, I think that their definitions are bizarre…”*

**R:** I agree. Still, I think it should be possible to find a more charitable interpretation. I am increasingly convinced that some disagreements, especially between philosophers and clinicians, are just misunderstandings based on different mental frameworks—not only with different ways of approaching the same questions but also different views on what it means to give these questions a satisfactory answer. This does not necessarily mean that these particular definitions are correct or even justifiable, but it means that we should (also) assess their validity/justification against the internal logic of functional attributions in medicine. What is this internal logic? That's the question I ask myself.

**C:** *“It is interesting to ask about the field within which we should address the question of function…”*

**R:** Your comment prompted me to adopt the role of the devil’s advocate and seek alternative interpretations. First, the distinction between “measurable on bedside” vs. “recordable by technology” reminded me of debates about theory and observation in science and whether the use of instruments to augment the human senses counts as an observation. Second, we could reverse the reasoning. Let’s suppose that Greer, Shemie, and colleagues make the following implicit assumption: only living organisms have functions. Based in it, we could argue that if brain dead patients are dead (and I am sure they truly believe it) and if, despite being dead, there is still osmoregulation and other hypothalamic activity, then these activities are not functional. These lines of argumentation are just two sketchy examples that could be further developed and improved. Personally, I find them unconvincing, but at least they make sense (kind of).

**C:** *“Finally, the notion that “brain functions are not merely considered as physiological mechanisms…”*

 I don't have a clear idea on this yet. I would need to think about it more.

**C:** *“And then second, a function as understood within the context of natural selection would not have any “intended” purpose, behaviorally manifested or not…”*

 Obviously, functions (and biological traits in general) don’t have intentions. The notions of intention, purpose, design, etc., are controversial in biology and are usually considered as informal ways of talking. The intended purpose of a biological trait (organ, structure, process, etc.), in the context of natural selection, is what this trait (an occurrence of it) is supposed to do according to the type it belongs to, and this type is constituted by its etiology. In other words, the sinoatrial node of a particular heart in a particular individual is supposed to preserve the heartbeat (and thus contribute to the circulation of blood) because it belongs to a type of cells (or group of cells) who, because they have had in the past (previous generations) the consequence of preserving the heartbeat, have contributed to the reproductive success of their ancestor organisms. In simpler words, the function of a trait is what this trait has been selected for. This is what, in the context of an etiological conception of functions (e.g. Larry Wright, Karen Neander, Ruth Garrett Millikan), we can consider as being the “intended purpose” of a trait, although there is nobody or nothing to actually intend it nor any actual purpose into it, but it is an easier way of talking.

 I have now put the expression “intended behavioural purpose” between quotation marks to indicate that it must not be taken literally.

**C:** *“On the dead donor rule…”*

**R:** Indeed, it is the dead donor rule that prohibits organ procurement, not the criterion itself, and I agree that both things must be independent. However, we can consider death criteria from at least two different perspectives. One is medical, the other is legal. Medical criteria are supposed to determine whether the patient is dead or not as a matter of fact in the biological realm (I am not sure whether this characterisation is accurate and, if so, whether I would agree with it, but let’s assume it), whereas legal criteria determine whether the patient is dead or not as a matter of law in the social and legal realms. Legal death allows social and legal practices beyond organ donation: autopsies, burial, mourning, inheritance, payment of life insurances, etc. Legal death and biological death do not necessarily coincide. For instance, an individual who has been missing for many years can be declared dead *in absentia*, without any actual proof of their death. This is a good policy because families and society as a whole need such a mechanism (although there may be false positives). Similarly, we could consider the UDDA (i.e. the current legal criteria of death, including the brain criterion) as good policy regardless of the issue of organ procurement.

**C:** *“Nair-Collins does not defend the brain criterion…”*

**R:** Indeed. I corrected the wording of the sentence to avoid any confusion: “As a consequence, some advocates of the brain criterion…, whereas critics…, or homeostasis (Nair-Collins 2018)”.
